# Essential Oils as Stress-Reducing Agents for Fish Aquaculture: A Review

**DOI:** 10.3389/fphys.2019.00785

**Published:** 2019-06-20

**Authors:** Carine de Freitas Souza, Matheus Dellaméa Baldissera, Bernardo Baldisserotto, Berta Maria Heinzmann, Juan Antonio Martos-Sitcha, Juan Miguel Mancera

**Affiliations:** ^1^Department of Physiology and Pharmacology, Universidade Federal de Santa Maria, Santa Maria, Brazil; ^2^Department of Microbiology and Parasitology, Universidade Federal de Santa Maria, Santa Maria, Brazil; ^3^Department of Industrial Pharmacy, Universidade Federal de Santa Maria, Santa Maria, Brazil; ^4^Department of Biology, Faculty of Marine and Environmental Sciences, Instituto Universitario de Investigación Marina, Campus de Excelencia Internacional del Mar, University of Cádiz, Cádiz, Spain

**Keywords:** aquaculture, natural compounds, fish stress, fish health, fish immundogy

## Abstract

In fish, stressful events initiate a hormone cascade along the hypothalamus-pituitary-interrenal and hypothalamus-sympathetic-chromaffin (HSC) axis to evoke several physiological reactions in order to orchestrate and maintain homeostasis. Several biotic and abiotic factors, as well as aquaculture procedures (handling, transport, or stocking density), activated stress system inducing negative effects on different physiological processes in fish (growth, reproduction, and immunity). In order to reduce these consequences, the use of essential oils (EOs) derived from plants has been the focus of aquaculture studies due to their diverse properties (e.g., anesthetic, antioxidant, and antimicrobial), which have been shown to reduce biochemical and endocrine alterations and, consequently, to improve the welfare status. Recently, several studies have shown that biogenic compounds isolated from different EOs present excellent biological activities, as well as the nanoencapsulated form of these EOs may potentiate their effects. Overall, EOs presented less side effects than synthetic compounds, but their stress-reducing efficacy is related to their chemical composition, concentration or chemotype used. In addition, their species-specific actions must be clearly established since they can act as stressors by themselves if their concentrations and chemotypes used are not suitable. For this reason, it is necessary to assess the effect of these natural compound mixtures in different fish species, from marine to freshwater, in order to find the ideal concentration range and the way for their administration to obtain the desired biological activity, without any undesired side effects. In this review, the main findings regarding the use of different EOs as stress reducers will be presented to highlight the most important issues related to their use to improve fish welfare in aquaculture.

## Introduction

The aquaculture industry deals with several stressful situations that can compromise the target species well-being, including handling, confinement, fertilization, transport, and other operations, from the hatchery to the final commercial stage (Ashley, 2007; Sampaio and Freire, 2016; Sneddon et al., 2016; Sánchez-Muros et al., 2017). Stress induced by such practices has long been suspected to cause mortality, affecting the success in fish production with the consequent economic loss for this sector. In addition, the impact of aquaculture-related stressors can also predispose fish to disease (Segner et al., 2012).

**FIGURE 1:**
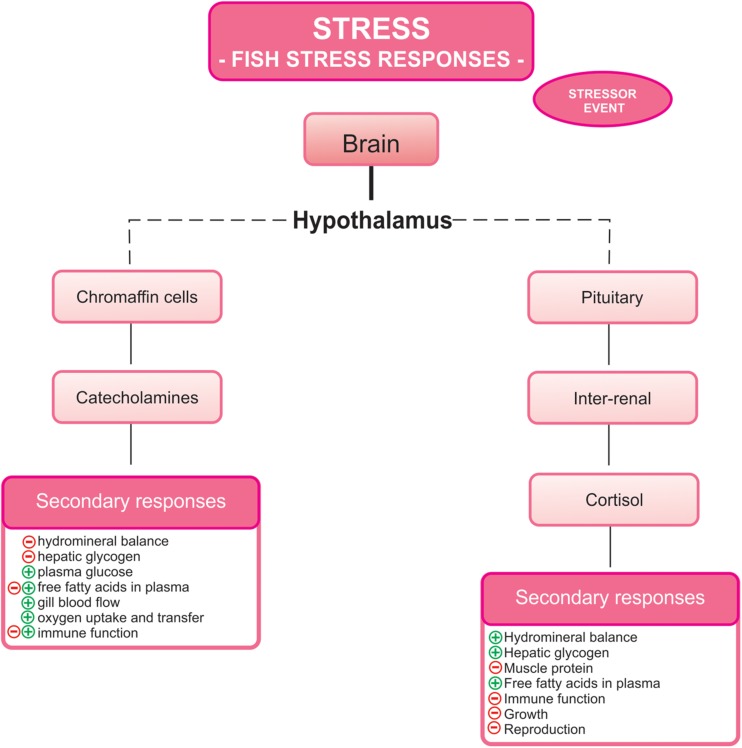
Responses to stress in fish. The activation of the hypothalamus-sympathetic-chromaffin and hypothalamus-pituitary-interrenal axes culminates in the release of catecholamines and cortisol, respectively. These hormones induced several stress secondary responses (modified from Wendelaar Bonga, 1997). (+) indicates activation and/or increase and (−) indicates inhibition and/or reduction.

Stress response is usually triggered by a wide range of physiological mechanisms in order to compensate the imbalances produced by the stressor and recover the homeostatic status of fish. The stress response is initiated and controlled by two hormonal systems, which lead to the production of catecholamines (such as adrenaline and noradrenaline, and their precursor dopamine) by the hypothalamus-sympathetic-chromaffin (HSC) axis, and corticosteroids (mainly cortisol) by the hypothalamus-pituitary-interrenal (HPI) axis (Wendelaar Bonga, 1997, 2011; Flik et al., 2006; Martos-Sitcha et al., 2014). Behavioral changes are used by the organism to overcome this situation, subsequently generating several responses to the stressor, including gene, metabolic, energetic, immune, endocrine, and neural changes (Schreck and Tort, 2016; Figure 1). Long-term consequences of repeated or prolonged stressful exposures are maladaptive by negatively affecting other necessary life functions, such as growth, development, disease resistance, behavior or reproduction and may even culminate in fish death (Wendelaar Bonga, 1997; Schreck and Tort, 2016). Therefore, several studies assessed the use of sedatives and anesthetics in order to find alternatives to minimize stress effects caused by the intense management practices in aquaculture (Ross and Ross, 2008; Neiffer and Stamper, 2009; Sneddon, 2012; Zahl et al., 2012). Recent excellent reviews have focussed on this topic (Hoseini et al., 2018; Priborsky and Velisek, 2018; Vanderzwalmen et al., 2018).

In addition, essential oils (EOs) derived from plants have been used in aquaculture studies because of their diverse properties that can improve health, growth and welfare of animals (Azambuja et al., 2011; Zeppenfeld et al., 2014; Souza et al., 2018a), as well as reduce stress processes (Saccol et al., 2016, 2018a; Souza et al., 2017b). There are reviews of the effects of EOs as sedatives, anesthetics, antioxidants, and antimicrobials (Cunha et al., 2018; Hoseini et al., 2018; Sutili et al., 2018). Hoseini et al. (2018) focused on stress-reducing effects, but only related to anesthetic effects of the EOs, while the other two reviews did not deal with this subject. Therefore, the aim of the present review is to discuss the possible mechanisms by which the different ways of applying EOs (waterborne and dietary exposure) may minimize stress responses induced by aquaculture procedures, as well as a future perspective of their use.

## Essential Oils

Essential oils extracted from plants contain compounds produced during plant secondary metabolism. They constitute one of the most important groups of raw materials for the food, hygiene, and cleaning products and pharmaceutical industries, perfumery, and others. They are complex mixtures of low molecular weight substances (Morais, 2009), with a wide variation in their chemical properties (Hussain et al., 2008). Several studies demonstrate that certain EOs, used as anesthetics and/or sedatives, reduced plasma cortisol levels and attenuated stress response (Cunha et al., 2010; Zeppenfeld et al., 2014; Souza et al., 2017b). Moreover, some EOs when added to therapeutic baths (concentrations lower than those that induce sedation) are able to prevent oxidative stress. The EO of *Melaleuca alternifolia*, for example, was also able to prevent the inhibition of splenic creatine kinase and pyruvate kinase activities caused by diseases (Baldissera et al., 2017c, 2018), thus demonstrating the possibility to be used as a stress-reducing agent in aquaculture practices. For example, anesthesia with the EO of *Lippia alba* (citral and linalool chemotypes) prevented plasma cortisol increase in silver catfish (*Rhamdia quelen*) exposed to air for biometry (Cunha et al., 2010; Souza et al., 2018a), and altered the expression of hormones and enzymes of the HPI axis, proving that it can directly influence this cascade (Souza et al., 2019). Furthermore, it is important to note that EOs have lipophilic properties and liposolubility, contributing to rapid dispersion through biological membranes, including the blood-brain barrier in the central nervous system (CNS), modulating brain function (Zahl et al., 2012; Manayi et al., 2016). It is known that several EOs exert their anesthetic effects by regulating the gamma-aminobutyric acid receptor complex (GABA), the main inhibitory neurotransmitter in the CNS (Bakkali et al., 2008; Chioca et al., 2013; Heldwein et al., 2014; Moreira et al., 2014; Raut and Karuppayil, 2015). Some EOs act in the benzodiazepine site of the GABA_A_ receptor, but others do not (Heldwein et al., 2012; Silva et al., 2012; Garlet et al., 2016; Bianchini et al., 2017; Santos et al., 2017a). In addition, deep anesthesia with *Cymbopogon nardus* EO promotes a conspicuous depression on muscle contraction power with loss of muscle tonus and transient cardiorespiratory depression (Barbas et al., 2017b).

### Definition

EOs are natural multicomponent systems of volatile, lipophilic, odoriferous and liquid substances, obtained from plant raw materials (Edris, 2007). The number of components of an EO generally ranges from 20 to 200, and they are named according to their concentration in the mixture, as (i) major constituents (from 20 to 95%), (ii) secondary constituents (1–20%), and (iii) trace components (below 1%). More than 3,000 distinct chemicals have been detected in EOs, with great variety of chemical structures (Sticher, 2015). Overall, they are classified as terpene hydrocarbons, simple and terpene alcohols, aldehydes, ketones, phenols, esters, ethers, oxides, peroxides, furans, organic acids, lactones, coumarins, or even sulfur compounds (Swamy et al., 2016).

### Chemical Composition, Extraction Methods, and Stability of Essential Oils

The genetic characteristics of the producing plant are the most important factors determining the chemical composition of an EO and, consequently, interfere with their biological/pharmacological activities. In addition, the chemical composition is generally specific for a given organ and characteristic of its stage of development, but edaphic and environmental conditions, as well as the extraction method used may cause significant variations (Figueiredo et al., 2008; Reyes-Jurado et al., 2015). Since chemical variability is often high among plants from their natural habitat, whenever possible, EOs should be obtained from cultivated plants that have genetic homogeneity, to ensure the consistency of the composition. However, it is also necessary to observe other aspects that influence the composition of an oil (Sticher, 2015), already cited above, trying to keep them constant.

The EO extraction methods are among the aspects that most affect the chemical characteristics. The main methods of EO extraction are steam distillation and its variants, cold pressing, supercritical fluid extraction, and solvent extraction (Luque de Castro et al., 1999; Capuzzo et al., 2013; Reyes-Jurado et al., 2015). Another variability factor is the occurrence of chemotypes or chemical races, which is often related to geographical variations (Figueiredo et al., 2008). This phenomenon is characterized by botanically identical plants which differ chemically (Aprotosoaie et al., 2017). Consideration should also be given to the relative low stability of EOs, which may undergo chemical changes mainly due to loss of volatile compounds, oxidation reactions and/or polymerization. However, various strategies can be observed to prevent their deterioration, including storage in small volume glass containers, maintaining them under low temperatures and protected from light. Further information on the stability of EOs can be obtained in Turek and Stintzing (2013).

## Effects of EOs During Different Stress Events

The interpretation of the general effects of EOs is somewhat complicated because only a few were tested in more than one fish species during different stress events. Furthermore, type and characteristics of stressors used may differ complicating the comparison between EOs. In addition, most studies were performed in freshwater species but not in marine species. Finally, other factors that hinder the comparisons are the existence of different chemotypes for the same plant and the variability of the composition of EOs even from the same chemotype.

### Stress-Preventing Effects During Handling Procedures

Exhaustive swimming during attempts to escape from the capture induces changes in physiological parameters, such as increase in glucose and lactate values, when compared to resting animals (Brown et al., 2008; Olsen et al., 2013). Furthermore, previous studies showed that intense swimming activity during chase and capture can be sufficient to compromise flesh quality (Cole et al., 2003) and reproduction (Pankhurst and Van der Kraak, 1997). The handling procedure may also include exposure of fish to air, a situation that requires a short-term orchestration of different endocrine players from both HSC and HPI axes (Wendelaar Bonga, 2011; Skrzynska et al., 2018).

Despite the purpose of using an anesthetic being to mitigate stress, a common observation is that the substance itself may pose as a stressor, thus activating the stress response mechanism (Thomas and Robertson, 1991; Sladky et al., 2001; Bolasina, 2006) and these situations have been observed in fish exposed to MS-222, metomidate, quinaldine sulfate, benzocaine, and phenoxyethanol (Thomas and Robertson, 1991; Tort et al., 2002; Wagner et al., 2002). In addition, several synthetic anesthetics are aversive to fish even at low concentrations (Readman et al., 2013). In this sense, an increasing attention has been focused on the use of plant extracts in fish anesthesia due to a wide range of beneficial health benefits, such as antioxidant, antimicrobial, stress-relieving and immune-promoting effects (Zahl et al., 2010; Hoseini, 2011; Zeppenfeld et al., 2014; Souza et al., 2018b). Moreover, the EOs of *Lippia alba* (chemotype linalool) and *Aloysia triphylla* are not aversive to fish (Bandeira Junior et al., 2018), indicating another advantage compared to synthetic anesthetics.

The use of EOs as sedatives or anesthetics to minimize possible damage to fish resulting from handling was recently demonstrated in several species (Cunha et al., 2010; Benovit et al., 2012; Tondolo et al., 2013; Toni et al., 2015; Hashimoto et al., 2016; Pedrazzani and Ostrenski Neto, 2016; Ribeiro et al., 2016; Barbas et al., 2017a, b; Fogliarini et al., 2017; Bodur et al., 2018a, b; Hoseini et al., 2018; Souza et al., 2018a, b). For minor procedures such as biometry and collection of blood samples, lower concentrations of EOs can be used that induce tranquilization and light sedation, in order to minimize stress and reduce plasma cortisol levels (Table 1). The recommended concentration of clove oil (extracted from *Syzygium aromaticum* or *Eugenia aromatica*) in handling processes is 10–30 mg⋅L^–1^ (Javahery et al., 2012). However, exposure seawater gilthead seabream (*Sparus aurata*) at concentration of 44.5 μL⋅L^–1^ before air exposure for blood collection increased plasma cortisol and/or glucose values compared to control fish (handled as the anesthetized fish) (Bressler and Ron, 2004). Previous anesthesia with 10 mg⋅L^–1^ clove oil did not change plasma cortisol and glucose levels of the seawater meager manipulated for weighing compared to control fish (handled as the anesthetized fish) (Barata et al., 2016). On the other hand, for seawater Senegal sole (*Solea senegalensis*) anesthesia with 1000 mg⋅L^–1^ clove oil demonstrated a good ability of clove oil to prevent cortisol, lactate and glucose increases pre-mortem (Ribas et al., 2007). In addition, studies demonstrated that the faster anesthesia induction with eugenol and clove oil, the lower the stress response provoked by these anesthetics (Hoseini and Nodeh, 2013; Mirghaed et al., 2018) thus, low concentrations of eugenol besides causing slow anesthetic induction may cause disturbances in plasma glucose levels and plasma cortisol.

**TABLE 1:** The use of essential oils (EOs) as stress-reducing agents in fish exposed to handling and air exposure.

**Essential oil**	**Major**	**Fish species**	**Stress/**	**Purpose/**	**Effect on fish**	**References**
**(EO)**	**compounds (%)**		**duration**	**concentration**	**physiology**	
*Aloysia triphylla*	Geranial (28.97)	*O. niloticus*	Air exposure/1 min	Anesthesia/300 μL⋅L^–1^	Prevents increase in plasma cortisol levels	Teixeira et al.,2017
	β-citral (20.78)	*R. quelen*	Handling	Diet supplemented with 2.0 mL per kg	Reduces plasmatic cortisol and lactate levels	Zeppenfeld et al.,2017
*Eucalyptus* sp.	1.8 cineole (80.84%)	*D. labrax*	Handling	Anesthesia/300 μL⋅L^–1^	Induces a stress response after 24 h of exposure, inducing plasma cortisol enhancement and up regulation of *hsp90* and *gr* gene expression in liver	Bodur et al.,2018b
*Lippia alba*	Linalool (55.26%)	*R. quelen*	Air exposure/1 min	Anesthesia and stress-reducing agent/100–500 mg⋅L^–1^	Reduces plasma cortisol levels	Cunha et al., 2010; Souza et al.,2018a
	β-linalool (50.56%)			Anesthesia/100–300 μL⋅L^–1^	Decreases TBARS and protein carbonyl levels in liver and kidney	Souza et al.,2018a
	Linalool (54.38%)		Handling and air exposure/1 min	Anesthesia/300 and 450 μL⋅L^–1^	Prevents Na^+^-K^+^-ATPase activity reduction due to handling.	Toni et al.,2014
	β-linalool (50.56%)			Anesthesia/100–300 μL⋅L^–1^	Decreases *slc6a2* and *crh* gene expression in brain.	Souza et al.,2019
	β-linalool (87.6%)	*S. aurata*	Persecution/1 min	Stress-reducing agent/ 35 μL⋅L^–1^	Increases *pomcb* expression in pituitary	Toni et al.,2015
*Lippia alba*	E-citral (29.84%)	*R. quelen*	Air exposure/1 min	Anesthesia/300 μL⋅L^–1^	Enhances protein carbonyl levels in liver and kidney	Souza et al.,2018a
					Increases s*lc6a2*, *crh, hsd20b, hspa12a, hsp90* gene expression in brain	Souza et al.,2019
	Geranial (25.4%)	*C. macropomum*	Rapid air exposure for biometry	Sedation and stress-reducing agent/50 and 100 μL⋅L^–1^	Increases plasma glucose and ammonia values.	Batista et al.,2018
*Hesperozygis ringens*	Pulegone (95.17%)	*R. quelen*	Handling and air exposure/1 min	Anesthesia/300 and 450 μL⋅L^–1^	Prevents Na^+^-K^+^-ATPase activity reduction caused by handling.	Toni et al.,2014
*Mentha spicata*	Carvone (28.4%)	*C. carpio*	Handling	Anesthesia/5 mL L^–1^	Reduces opercular rate and decreases plasma glucose levels after recovery	Roohi and Imanpoor,2015
*Ocimum gratissimum*	Eugenol (43.3%)	*B. amazonicus*	Handling/fish transfer from cage to buckets and then back to cage	Anesthesia and stress-reducing agent/20–60 mg⋅L^–1^	Increases plasma glucose and lactate/ammonia values only at 60 mg⋅L^–1^	Ribeiro et al.,2016
	Eugenol (73.6%)	*R. quelen*	Handling	Anesthesia/70 and 300 mg⋅L^–1^	Enhances plasma glucose levels	Silva et al.,2012
*Ocimum americanum*	1,8-cineole (21.0%)	*R. quelen*	Air exposure/1 min	Anesthesia/300 and 500 mg⋅L^–1^	Prevents plasma cortisol increase and Na^+^ loss	Silva et al.,2015
*Origanum* sp.	Carvacrol (78.16%)	*D. labrax*	Handling	Anesthesia/50 μL⋅L^–1^	Decreases stress-related genes (*cyb11b1* and *star*) expression in head kidney	Bodur et al.,2018b
*Syzygium aromaticum* (clove oil)	Eugenol	*C. carpio*	Handling	Anesthesia/700 mg⋅L^–1^	Increased plasma cortisol and glucose levels	Hoseini and Nodeh,2013
		*R. quelen*	Handling	Anesthesia/50 mg⋅L^–1^	Decrease plasma cortisol and glucose levels	Cunha et al.,2010
		*S. aurata* and *O. mykiss*	Handling	Anesthesia/50–200 μL⋅L^–1^	Does not prevent plasma cortisol increase caused by handling	Tort et al.,2002

The effect of the EOs may also change according to the species and the type of application. For example, Nile tilapia anesthetized with 300 μL⋅L^–1^ of the EO of *A. triphylla* and then exposed to air for 1 min decreased plasma cortisol levels respect to control fish (handled as the anesthetized fish) (Teixeira et al., 2017). However, silver catfish anesthetized with 135 and 180 mg⋅L^–1^ and submitted to the same procedure did not change plasma cortisol values compared to control fish (Gressler et al., 2014). Interestingly, silver catfish fed diet supplemented with 2.0 mL per kg of this EO for 21 days and handled for blood collection presented lower plasma cortisol and lactate levels, but not glucose, than fish that received control feed and were submitted to the same procedure (Zeppenfeld et al., 2017; Table 1). However, the same dietary dose of this EO did not change whole body cortisol content in zebrafish handled for biometry (Zago et al., 2018).

The EO of *Ocimum americanum* used as anesthetic prevented plasma cortisol increase and Na^+^ loss induced by aerial exposure (1 min) in silver catfish compared to control fish (handled as the anesthetized fish) (Silva et al., 2015). However, the EO of a plant from the same genus, but different species (*O. gratissimum*, with eugenol and 1,8-cineole as the main compounds), did not prevent this cortisol enhancement from air exposure. In addition, hyperglycemia was verified in fish exposed to 70 and 300 mg⋅L^–1^ at 1 and 4 h after handling, indicating no effect on attenuation of stress axis activation (Silva et al., 2015). Matrinxãs (*Brycon amazonicus*) transferred from cage nets to 20 L buckets with 60 mg⋅L^–1^ of the EO of *O. gratissimum* for 10 min also increased plasma glucose levels and did not prevent plasma lactate enhancement compared to control fish (handled as the anesthetized fish) (Ribeiro et al., 2016; Table 1). Consequently, even having eugenol as one of the main compounds, the efficacy of these two EOs to reduce stress is species-specific.

Anesthesia of silver catfish with the EO *L. alba* (300 μL⋅L^–1^) before handling and air exposure (1 min) prevented plasma cortisol increase observed 1 and 4 h later in control fish (handled as the anesthetized fish) (Cunha et al., 2010). In addition, both linalool and citral chemotypes of this EO (300 μL⋅L^–1^) reduced plasma cortisol through anesthesia and the first 10 min of recovery compared to control fish (handled as the anesthetized fish) (Souza et al., 2017b). Finally, individuals of this species anesthetized with linalool chemotype of this EO (100 and 300 μL⋅L^–1^) decreased, at 4 h after anesthesia and air exposure for biometry and compared to control fish, expression of genes directly related to stress: corticotropin releasing hormone (*crh*), and solute carrier family 6 (neurotransmitter transporter, noradrenalin) member 2 (*slc6a2*) (Souza et al., 2019). However, the citral chemotype is stressful for silver catfish because there was an up regulation of s*lc6a2*, *crh*, 20β-hydroxysteroid dehydrogenase (*hsd20b*) and heat shock proteins 70 member 12 (*hspa12a*) and heat shock protein 90 (*hsp90*) (Souza et al., 2019; Table 1).

Similarly to other EOs, the efficacy of the linalool chemotype of the *L. alba* EO as a stress reducing agent can change with the concentration and/or species. So, 200 μL⋅L^–1^ did not change plasma cortisol levels in the hybrid tambacu (*Piaractus mesopotamicus* × *Colossoma macropomum*) subjected to handling compared to control fish (handled as the anesthetized fish) (Sena et al., 2016). However, anesthesia of tambaqui (*Colossoma macropomum*) with 50 and 100 μL⋅L^–1^ EO of this plant, but of the chemotype citral, did not prevent plasma glucose and ammonia enhancement due to rapid air exposure for biometry (Batista et al., 2018; Table 1).

Others EOs from different plants showed variable effects depending on the species tested. Anesthesia with the EOs of *Hesperozygis ringens* (137–277 μL⋅L^–1^) and *Ocotea acutifolia* (300 μL⋅L^–1^) did not change blood glucose levels in silver catfish (Silva et al., 2013). The EOs of *Myrcia sylvatica* and *Curcuma longa* anesthetized matrinxã and reduced plasma cortisol levels compared to control fish (handled as the fish exposed to EOs) (Saccol et al., 2017). Finally, the use of 50 μL⋅L^–1^ of oregano EO (*Origanum* sp.) as anesthetic decreased the expression of steroidogenic genes at 2 and 24 h compared to clove oil, demonstrating the efficiency of oregano EO in reducing secondary stress in European sea bass (Bodur et al., 2018a, b; Table 1).

**FIGURE 2:**
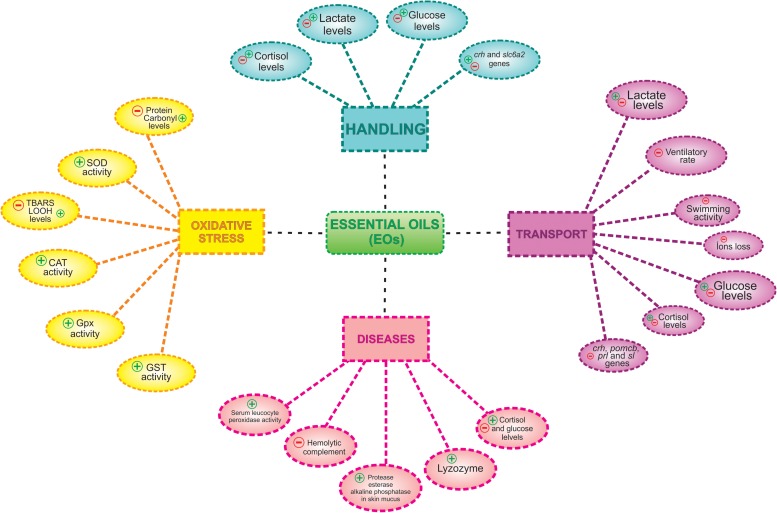
Effects of essential oils (EOs) on physiological changes induced by aquaculture procedures, handling, transport, and diseases. (+) indicates activation and/or increase and (−) indicates inhibition and/or reduction.

In the marine dusky grouper the use of 50 and 300 μL⋅L^–1^ of the EO of *Aloysia polystachya* for, respectively, sedation and anesthesia, did not affect plasma glucose, Na^+^, and Cl^–^ levels compared to control fish (handled as the anesthetized fish) (Fogliarini et al., 2017; Figure 2).

Usually, EOs are applied in water prior to handling. However, it would be interesting to test the efficacy of EOs if other means of administration were used. In this sense, dietary addition (0.5–2.0 mL⋅kg^–1^) of cinnamon (*Cinnamomun* sp.) EO did not alter plasma cortisol and glucose levels of Nile tilapia exposed to air for 3 min compared to control fish, which was fed diet without this EO and subjected to the same procedure) (Santos et al., 2016).

### Stress-Preventing Effects During Transport Procedures

Live transportation of larvae, juvenile or broodstock is one of the major causes of stress in fish due to the capture, packing, high loading density, changes in water quality, the transport itself, unloading, and the final storage (Harmon, 2009; Sampaio and Freire, 2016). In all transport systems, including simple closed systems, such as sealed plastic bags partly filled with water and oxygen, homeostasis of the transported fish is mainly challenged by the build-up of ammonia (NH_3_ and NH_4_^+^) due to excretion, and carbon dioxide (CO_2_) from respiration (Lim et al., 2003; Paterson et al., 2003; Becker et al., 2016). These events cause changes in pH (Golombieski et al., 2003), which may alter fish metabolism and induce activation of the stress system. So, transportation can lead to a number of physiological responses such as catecholamines and corticosteroids release as well as blood glucose levels enhancement (Pottinger, 2008; Zeppenfeld et al., 2014). In addition, transport significantly decreased corticotrophin-releasing hormone (*crh*) and urotensin I (*ui*) expression levels in the brain of *Coilia nasus* and induced oxidative stress (Du et al., 2015). Furthermore, stress by transport induced an osmotic imbalance with ion loss in freshwater fish, showing a good correlation with transport stress (Becker et al., 2013, 2016; Parodi et al., 2014; Zeppenfeld et al., 2014; Garcia et al., 2015; Ostrensky et al., 2016; Teixeira et al., 2018; Table 2). Thus, during or prior to transport, sedation of the fish is desirable to reduce or prevent stress.

**TABLE 2:** The use of essential oils (EOs) as stress-reducing agents in fish transport.

**Essential**	**Major**	**Fish species**	**Stress/**	**Purpose/**	**Effect on fish**	**References**
**oil (EO)**	**compounds (%)**		**duration**	**concentration**	**physiology**	
*Aloysia gratissima*	NA	*P. orbignyanus*	Transport in plastic bags/7 h	Anesthetic/90 mg⋅L^–1^	Increases plasma glucose values and mortality	Benovit et al.,2012
*A. triphylla*	E-citral (42.30)	*R. quelen*	Transport/4 h	Sedation/40 and 50 μL⋅L^–1^	Reduces ammonia excretion and plasma cortisol levels	Zeppenfeld et al.,2014
	α-citral (20.41)	*L. alexandri*	Transport/4 h	Sedative and stress-reducing agent/25 μL⋅L^–1^	Decreases total ammonia nitrogen levels and ventilatory frequency.	Becker et al.,2017
*Curcuma longa*	β-selinene (9.96)	*B. amazonicus*	Transport simulation/360 min	Sedation/40 μL⋅L^–1^	Reduces plasma cortisol levels and preventing a stress response as well as excess of reactive oxygen species formation.	Saccol et al.,2017
*L. alba*	Linalool^*^	*R. quelen*	Transport/5, 6, 7 h	Slight sedation/10–20 μ⋅L^–1^	Improves redox state	Azambuja et al.,2011
			Transport/4 h	Stress- reducing agent/1.5 and 3.0 μL⋅L^–1^	Reduces net Na^+^, Cl**^–^**, and K^+^ loss	Becker et al.,2012
			Transport/6 h	Sedation/30–40 μL^–1^	Induces oxidative stress	Salbego et al.,2014
	Linalool (47.66)	Tambacu (*P. mesopotamicus × C. macropomum*)	Transport (8 h) and handling (0, 1 and 4 h)	Stress- reducing agent and anesthesia/10–200 μL⋅L^–1^	Prevents plasma cortisol levels enhancement	Sena et al.,2016
		*A. regius*	Simulated transport/4 h	Anesthetic/12 mg⋅L^–1^	Increases plasma cortisol values	Cárdenas et al.,2016
*Myrcia sylvatica*	β-phellandrene (31.48)	*B. amazonicus*	Transport simulation/6 h	Sedation/10 μL⋅L^–1^	Decreases plasma cortisol levels as well as prevents stress response and excess of reactive oxygen species formation	Saccol et al.,2017
	β-phellandrene (31.48)	*R. quelen*	Pre-transport handling/transport (6 h)	Stress-reducing agent	Reduces plasma cortisol and lactate levels as well as increases *crh*, *pomcb*, *prolactin*, and *somatolactin* gene expression in brain	Saccol et al.,2018b
*Nectandra grandiflora*	NA	*C. macropomum*	Transport/2, 6, and 10 h	Sedation and stress-reducing agent/30 μL⋅L^–1^	Enhances protection against oxidative damage mainly in muscle and gills	Barbas et al.,2017b
*N. megapotamica*	bicyclogermacrene (34.6)	*C. parallelus*	Transport/10 h	Stress-reducing agent/30 and 300 μL⋅L^–1^	Increases plasma glucose and lactate levels.	Tondolo et al.,2013
*Syzygium aromaticum* (clove oil)	Eugenol	*O. niloticus*	Transport simulation/3.5 h	Sedation/20 μL⋅L^–1^	Decrease plasma cortisol and glucose levels	Navarro et al.,2016
		*O. aureus*		3 mg⋅L^–1^	Increase in plasmatic cortisol levels	Akar,2011
				and 1 mg⋅L^–1^	Decrease plasmatic cortisol levels	
		*S. aurata*	Transport simulation/6 h	Sedation/2.5 mg⋅L^–1^	Increase in plasmatic cortisol levels and expression of *star* and *cyp11b1* genes in head kidney, interferes with fish antioxidant status	Jerez-Cepa et al., 2019; Teles et al.,2019

NA, data not available, ^*^ percentage not stated.

The EO of *L. alba* (linalool chemotype) at 10 or 20 μL⋅L^–1^ added to the transport water of silver catfish reduced net Na^+^, Cl**^–^**, and K^+^ loss compared to control fish subjected to the same procedure (Becker et al., 2012). The same chemotype of this EO (10 μL⋅L^–1^) prevented plasma cortisol (but not glucose) increase provoked by transport in tambacu (Sena et al., 2016; Table 2). A similar concentration of this EO (15 μL⋅L^–1^) inhibited plasma glucose enhancement in slender seahorses (*Hippocampus reidi*) transported in plastic bags (Cunha et al., 2011). However, meager submitted to simulated transport with 12 mg⋅L^–1^ of the EO of *L. alba* (linalool chemotype) presented higher plasma cortisol levels compared to control fish subjected to the same procedure (Cárdenas et al., 2016; Table 2). The transport of common carp in freshwater with 50–200 mg⋅L^–1^ linalool decreased ammonia excretion, but increased serum cortisol, glucose and urea values and did not prevent the decrease of serum Na^+^ and Cl**^–^** provoked by transport compared to control fish submitted to the same procedure (Mazandarani et al., 2017). Recent studies showed that EO of *L. alba* (chemotype linalool) at 5–10 μL⋅L^–1^ reduced swimming activity of the black piranha, *Serrasalmus rhombeus*, compared to control fish subjected to the same procedure and may also be considered as options for the transport of this species (Almeida et al., 2018). These results obtained so far with the use of the EO of *L. alba* (linalool chemotype) and linalool for the transport of fish do not lead to the conclusion whether the differences are related to species, concentrations, or water quality (fresh and seawater).

The EO of *A. triphylla* has also been tested for transport procedures in freshwater and marine species. The use of this EO (40 and 50 μL⋅L^–1^) during a 6 h transport reduced net ion loss (Parodi et al., 2014), ammonia excretion and plasma cortisol levels in silver catfish compared to control fish submitted to the same procedure (Zeppenfeld et al., 2014). Plasma cortisol enhancement in fat snook due to transport was reduced by adding 20 μL⋅L^–1^ of this EO to the water (Parodi et al., 2016), whereas 30 μL⋅L^–1^ decreased ventilatory rate, ion loss and plasma glucose levels, with no effects preventing cortisol increase in Nile tilapia (Teixeira et al., 2018), both compared to their respective control fish subjected to the same procedure. In addition, this EO (25 μL⋅L^–1^) also decreased ventilatory rate of pacamã, *Lophiosilurus alexandri*, as well as ammonia excretion during 4 h transport (Becker et al., 2017; Table 2), also reducing the swimming activity of black piranha at concentration of 10 μL⋅L^–1^ (Almeida et al., 2018) both compared to their respective control fish subjected to the same procedure. Overall, this EO seems to be effective in reducing stress of transport in fish, irrespective of whether they are fresh- or seawater-adapted.

Transport of the Brazilian flounder for 7 h with the EOs of *Aloysia gratissima* (90 mg⋅L^–1^) and *Ocimum gratissimum* (10–20 mg⋅L^–1^) increased blood glucose levels compared to control fish subjected to the same procedure. In addition, the EO of *A. gratissima* induced mortality. Consequently, both EOs are not effective in transport of this species (Benovit et al., 2012). Moreover, the EO of *Nectandra megapotamica* at 15 or 30 μL⋅L^–1^ was not able to prevent the stress of transport in fat snook because it did not prevent deterioration in water quality and the post-transport mortality compared to control fish subjected to the same procedure (Tondolo et al., 2013; Table 2). Studies of the effect of these EOs in freshwater species are still lacking. Simulated transport (6 h) of gilthead seabream with clove oil at 2.5 mg⋅L^–1^ increased plasma cortisol levels and expression of head kidney gene-expressions related to cortisol production (*star* and *cyp11b1*) and stimulated amino acids catabolism (Jerez-Cepa et al., 2019). The gill’s mRNA levels of *gst3*, a target gene related with the antioxidant response and cell-tissue repair, enhanced after transportation with clove oil, returning to control values after recovery. However, the rest of transcripts assessed (*gpx1*, *cat*, *gr*, *mt*, and *hsp70*) related to these responses did not present any alteration (Teles et al., 2019; Table 2). Authors proposed that alternative concentrations of this EO should be tested for the transport of gilthead seabream.

Transport of silver catfish with the EO of *Myrcia sylvatica* (25 or 35 μL⋅L^–1^) reduced plasma cortisol and lactate levels, concomitantly with the decrease in the gene expression of *crh* and proopiomelanocortin b (*pomcb*), *prolactin* and *somatolactin* mRNAs compared to control fish without EO administration and subjected to the same procedure (Saccol et al., 2018b). Exposure of silver catfish to the EOs of *Citrus × aurantium*, *Citrus × latifolia* (50–100 μL⋅L^–1^), increased ventilatory frequency, but reduced ion loss and ammonia excretion compared to control fish subjected to the same procedure (Lopes et al., 2018). The EOs of *Cunila galioides* (25 and 50 μL⋅L^–1^) and *Origanum majorana* (100 μL⋅L^–1^) also decreased ion loss (Cunha et al., 2017) and *Lippia origanoides* (5–10 μL⋅L^–1^) reduced ventilatory frequency in silver catfish after 6 and/or 8 h of exposure (Becker et al., 2018) both compared to their respective control fish subjected to the same procedure. For instance, these EOs apparently reduced stress and may also be useful for fish transport (Figure 2).

### Stress-Preventing Effects During High Stocking Density

Recent studies demonstrated that EOs are capable of mitigating or preventing stress caused by different stocking densities. Dietary addition of 0.50 mL per kg EO *L. alba* (linalool chemotype) prevented the increase in cortisol levels in silver catfish submitted to a stressful condition of high stocking density (Souza et al., 2015). Similarly, a diet supplemented with *M. sylvatica* EO (2.0 mL per kg) for 90 days reduced cortisol levels in gilthead seabream after 22 days held at high stocking density (40 kg m**^–^**^3^) (Saccol et al., 2018a). These results may stimulate new studies on the influence of EOs on stress reduction caused by the different stocking densities of fish in culture systems.

### Regulation of Oxidative Stress

Pro-oxidant compounds are the oxygen reactive species (ROS) that can damage DNA, proteins and lipids (Evans and Halliwell, 1999; Morel and Barouki, 1999), whereas the antioxidants are any substance that can prevent or reduce the oxidation of a target molecule (Halliwell and Gutteridge, 2007). To maintain homeostasis, fish eliminate ROS to counteract oxidative stress and prevent or repair oxidative damage by antioxidant defense system, which includes antioxidant enzymes such as superoxide dismutase (SOD), catalase (CAT), glutathione peroxidase (GPx), glutathione-S-transferase (GST), and also some non-enzymatic antioxidants such as non-protein thiol (NPSH) (Tu et al., 2012; Birnie-Gauvin et al., 2017), in order to avoid lipid peroxidation, and carbonylation of proteins, usually evaluated by the biomarkers thiobarbituric acid-reactive substances (TBARS) and carbonyl protein (PC), respectively. In this sense, the presence of ROS in the cells triggers biochemical reactions that will culminate in decreased cellular function due to oxidative damage caused in proteins, carbohydrates and lipids, which can lead to apoptosis and accumulation of oxidized molecular aggregates. Normally, an oxidative stress frame is triggered due to an imbalance between the oxidant and the antioxidant production, detected by low concentrations of antioxidant enzymes and higher prooxidant levels, culminating with a lower immune system response (Biller and Takahashi, 2018).

Several EOs, when used as anesthetics or sedative for manipulation and/or transport, exhibit antioxidant capacity (Tables 1, 2). This antioxidant activity is also observed when the EOs are used at concentrations lower than those that induce sedation and as dietary supplements (Tables 1–3). Thus, the antioxidant effects of several EOs are not only due to the possibility of lower ROS production when fish are sedated or anesthetized.

**TABLE 3:** The use of essential oils (EOs) as stress-reducing agents in fish diseases.

**Essential**	**Major**	**Fish species**	**Stress/**	**Purpose/**	**Effect on fish**	**References**
**oil (EO)**	**compounds (%)**		**duration**	**concentration**	**physiology**	
*A. triphylla*	β-citral (20.78)	*R. quelen*	Infection by *A. hydrophyla*	Diet supplemented/2.0 mL per kg diet	Improves survival and decreases the total count of leukocytes, lymphocytes, and neutrophils	Santos et al.,2017b
*Citrus limon*	Limonene (54.4)	*O. mossambicus*	Infection by *E. tarda*	Diet supplemented/0.5, 0.75, and 1%	Enhances non-specific immune parameters and decrease mortality rate	Baba et al.,2016
*H. ringens*	Pulegone (96.63)	*R. quelen*	Infection by *A. hydrophyla*	Preventive baths/20 mg⋅L^–1^	Increases survival	Sutili et al.,2015
*Melaleuca alternifolia*	Terpinen-4-ol (27.15)	*R. quelen*	Infection by *A. hydrophyla*	Preventive baths/50 μL⋅L^–1^	Increases the non-specific immune system and prevents oxidative damage	Baldissera et al.,2017a
				Preventive baths/50 μL⋅L^–1^	Prevents alterations to purinergic enzymes and ameliorates the innate immune response.	Baldissera et al.,2017b
	Terpinen-4-ol (41.98)		Infection by *P. aeruginosas*	Preventive baths/50 μL⋅L^–1^	Ameliorates the hepatic antioxidant/oxidant status	Souza et al.,2017a
*Ocimum gratissimum*	1,8-cineole (40.4)	*O. niloticus*	Infection by *S. agalactiae*	Diet supplemented during 55 days/0.5, 1.0, and 1.5%	Improves growth, immune responses and disease resistance	Brum et al.,2017
*O. americanum*	β-linalool (46.6)	*R. quelen*	Infection by *A. hydrophyla*	Preventive baths/20 mg⋅L^–1^	Increases survival	Sutili et al.,2015
*Zataria multiflora*	NA	*C. carpio*	Low temperature and challenge by *A. hydrophyla*	Diet supplemented/30 and 60 mg⋅L^–1^	Enhances immune system	Soltani et al.,2010
*Zingiber officinale*	Geranial (24.0)	*O. niloticus*	Infection by *S. agalactiae*	Diet supplemented during 55 days/0.5 and 1.0%	Improves immune responses and disease resistance	Brum et al.,2017
*Syzygium aromaticum* (clove oil)	Eugenol	*R. quelen*	Infection by *A. hydrophyla*	Baths (5 and 10 mg⋅L^–1^)	Promoted the survival but did not change phagocytic activity, production of superoxide anion, serum hemolytic activity, and hematology.	Sutili et al.,2014

*NA, data not available.*

We highlight the EO of *L. alba* because it presents different responses to oxidative stress, which varies according to its concentration, chemotype, and fish species. For example, the addition of this EO (10–20 μ⋅L^–1^, linalool chemotype) during transport suppressed oxidative stress in silver catfish (Azambuja et al., 2011). However, this EO (linalool chemotype) at higher concentrations (30–40 μ⋅L^–1^) induced physiological and oxidative stress in silver catfish and gilthead sea bream (Salbego et al., 2014; Toni et al., 2015). On the one hand, silver catfish anesthetized with the chemotypes linalool and citral did not increase renal and hepatic thiobarbituric acid reactive species levels after anesthesia, avoiding lipid damage. On the other hand, fish anesthetized with the citral chemotype showed higher protein carbonylation levels, superoxide dismutase, catalase, and glutathione S-transferase activities as well as non-protein thiol group values in both tissues compared to controls. Therefore, the EO of both chemotypes present antioxidant capacity, but anesthesia with higher concentrations of the linalool chemotype does not cause damage to lipids or proteins, being more effective in anesthetizing silver catfish (Souza et al., 2018a).

Interestingly, anesthesia of silver catfish with the EO of *A. triphylla* (135 and 180 mg⋅L^–1^), whose main compound is citral, was capable of preventing the formation of lipid peroxides in the liver, possibly due to the increase of catalase and glutathione-S-transferase activities (Gressler et al., 2014). Similarly, anesthesia and sedation of tambaqui with the EOs of *M. sylvatica* and *C. longa* resulted in lower levels of lipid peroxidation and higher activity of antioxidant enzymes (superoxide dismutase, catalase, glutathione peroxidase, glutathione reductase, and glutathione-S-transferase), the content of non-protein thiols and total reactive antioxidant potential in several tissues (brain, liver, gills, and kidney) compared to control (Saccol et al., 2016). Anesthesia of common carp with 30 mg⋅L^–1^ clove oil did not alter TBARS or carbonyl protein levels in several tissues, but reduced superoxide dismutase in the brain as well as glutathione reductase and glutathione peroxidase in the brain and gills (Velisek et al., 2011).

In addition, the use of EOs in water during transport is also useful in order to activate the antioxidant defense system. Silver catfish transported with the EOs of *L. alba* (chemotype linalool) (10 and 20 μL⋅L^–1^) and *A. triphylla* (30 and 40 μL⋅L^–1^) in the transport water showed improvement in the redox state (Azambuja et al., 2011; Zeppenfeld et al., 2014; Salbego et al., 2017). However, this effect is dependent on the concentrations used. So, specimens treated with the EO of *L. alba* (chemotype linalool) prior to transport (200 μL⋅L^–1^ for 3 min) and transported for 6 h (with 30 or 40 μL⋅L^–1^) decreased significantly hepatic catalase, glutathione-S-transferase, glutathione peroxidase, non-protein thiol groups, and ascorbic acid levels compared to the control group (Salbego et al., 2014). This study also revealed that hepatic TBARS, protein oxidation levels, and the lipid peroxidation/catalase+glutathione peroxidase ratio were significantly higher in fish transported with both concentrations of this EO, indicating the existence of oxidative stress at hepatic level.

Exposure to EO of *Melaleuca alternifolia* (25 μL⋅L^–1^ – light sedation) for 6 h decreased hepatic TBARS levels followed by an increase in glutathione-S-transferase activity (Souza et al., 2018b). Finally, a sedative concentration (30 μL⋅L^–1^) of *N. grandiflora* EO enhanced protection against oxidative damage mainly in muscle and gills of tambaqui transported for up to 10 h (Barbas et al., 2017b; Figure 2).

The dietary supplementation for 20–60 days with different EOs usually improved oxidative status of freshwater fish. Channel catfish (*Ictalurus punctatus*) fed with 0.5 mL per kg EO of *Origanum vulgare* increased plasma lysozyme, catalase, and superoxide dismutase compared to fish fed a control diet (Zheng et al., 2009). The dietary addition of the EOs of *Cymbopogon citratus* (0.2 g per kg) and *Pelargonium graveolens* (0.4 g per kg) enhanced catalase and lysozyme activities and glutathione reductase content and reduced malondialdehyde from the whole body of the Nile tilapia compared to the basal diet (Al-Sagheer et al., 2018). Silver catfish fed 2.0 mL per kg EO of *A. triphylla* presented lower TBARS, lipid hydroperoxide, superoxide dismutase, catalase, and non-protein thiols in the brain, liver, and muscle of fish fed a control diet (Zeppenfeld et al., 2017). The same species fed diets supplemented with EO of *L. alba* (linalool chemotype, around 0.5–2.0 mL per kg) stimulated superoxide dismutase, catalase and glutathione peroxidase activities and non-protein thiols content, whereas reduced lipoperoxidation and TBARS in several organs compared to those fed a control diet (Saccol et al., 2013). Finally, hepatic superoxide dismutase and glutathione peroxidase activities enhanced while catalase, glutathione reductase, glutathione-S-transferase and malondialdehyde decreased in rainbow trout fed 0.5–1.0 g per kg diet of EOs of *Salvia officinalis*, *Mentha spicata*, and *Thymus vulgaris* compared to that of the control-diet fed group (Sönmez et al., 2015).

### Regulation of Immune System and/or Bactericidal Effects

The negative influence of stress on the immune system is well documented (Tort et al., 2003; Tort, 2011). In this regard, there has been a trend in the use of EOs for improving immune responses and disease resistance in fish (Bulfon et al., 2015). Often, the immunostimulants are administered as immersion or food additives, usually improving the innate (non-specific) defense mechanisms, increasing resistance to specific pathogens and promoting a recovery from immunosuppression states caused by stress (Sakai, 1999; Barman et al., 2013).

Some EOs used as anesthetics were also related as good immunostimulants for fish (Table 3). Anesthesia of gilthead seabream with 44.5 mg⋅L^–1^ clove oil did not change serum lysozyme activity, respiratory burst, and pinocytosis activity of head kidney (Bressler and Ron, 2004), but a concentration of 55 mg⋅L^–1^ increased serum leucocyte peroxidase activity and decreased hemolytic complement activity, which led to the conclusion that this EO did not change immune response in this species (Bahi et al., 2018). However, rainbow trout anesthetized with 25 mg⋅L^–1^ clove oil enhanced lysozyme and bactericidal activity, as well as protease, esterase, and alkaline phosphatase activities in the skin mucus, indicating potentiated skin mucosal immunity (Soltanian et al., 2018). Similarly, preventive baths with the EOs of *H. ringens* and *O. americanum* (20 mg⋅L^–1^) for 5 days increased survival in silver catfish infected with *A. hydrophila* (Sutili et al., 2015). Some EOs can have antimicrobial effects and, at the same time, act as immunostimulants. This is supported by the effects of the EO of *Melaleuca alternifolia*, which showed an antimicrobial effect against *A. hydrophila*, increased the non-specific immune system and prevented oxidative damage in silver catfish in fish exposed to 50 μL⋅L^–1^ for 7 days prior to infection (Baldissera et al., 2017a; Table 3).

The EOs may also have an immunostimulatory effect when added to the diet. For example, diet supplemented with the EO of *Zataria multiflora* enhanced common carp immunity to some extent even though fish could not express their potential immunity during stress caused by low temperature (Soltani et al., 2010). After 35 days of supplementation with EO *O. gratissimum* (0.5%), the Nile tilapia presented the lowest value of hematocrit and the highest numbers of neutrophils at 35 days. It can be inferred that this reduction is due to an energy offset for production of defense cells – in this case, neutrophils (Brum et al., 2017; Table 3). Adel et al. (2015) also observed a significant increase in neutrophils in the cyprinid kutum (*Rutilus frisii*) after dietary supplementation with the EO of *Mentha piperita*, asserting that there was stimulation of the innate immune response. However in spite of improving survival of silver catfish after *A. hydrophila* infection, the addition of 2.0 mL per kg diet of EO of *A. triphylla* showed an immunosuppressive activity because the total count of leukocytes, lymphocytes and neutrophils decreased (Santos et al., 2017b). In addition, supplementing the diet with EO of *Citrus limon* peels (0.5, 0.75, and 1%) improve non-specific immune parameters and decreased mortality rates in the Mozambique tilapia *Oreochromis mossambicus* challenged with *Edwardsiella tarda* (Baba et al., 2016; Table 3). These results indicate that diets enriched with EOs often present promising results for fish, and some EOs may even aid in improving the immune system as well as helping to prevent disease outbreaks in aquaculture systems.

## Conclusion and Future Perspectives on the Use of EOs in Aquaculture

Some EOs demonstrated advantages when used as sedatives/anesthetics compared to synthetic compounds because they are not aversive to fish, and reduce the stress responses related to handling and transport. On the other hand, their effects may vary according to plant variables such as chemotype, place of collection, climate, anatomical part from which the EO was extracted, altering significantly their composition and consequently their effects. Consequently, EOs should be obtained from cultivated plants that have genetic homogeneity, to ensure the consistency of their composition. Another perspective to be followed by future studies and by the pharmaceutical industry would be isolating/purifying the active compounds present in

the EOs to obtain more refined products that combine their beneficial effects with a better control of composition and maybe avoid undesired effects of uncontrolled compounds. Some isolated compounds, as linalool, have proportional anesthetic effect to the EO of *L. alba* chemotype linalool in silver catfish, but eugenol, which is the main compound of *O. gratissimum*, presents a narrower safety range that this EO. Additional studies comparing the effects and side effects of EOs and their isolated compounds are necessary to evaluate the advantages of using a given EO or its main compound(s). In addition, it is important to emphasize that the use of different concentrations of the EOs and the different fish species used can alter behavioral and physiological responses, as observed in synthetic anesthetics. As explained in a previous section, the stress-reducing effect of eugenol is better the faster the anesthesia induction. Is this relationship concentration/time to induce anesthesia/stress-reducing effect similar for EOs and their isolated compounds? Could this possible relationship explain, at least partially, the species-specific effects of the EOs studied so far?

Most studies show that EO also have promising potential for maintaining and promoting health, as well as preventing and potentially treating some diseases, ameliorating growth and antioxidant status. However, besides the high volatility, EOs can easily decompose, owing to direct exposure to heat, humidity, light, or oxygen, which would imply the loss of their effectiveness (Turek and Stintzing, 2013). Thus, it is interesting to develop technology to protect EOs when exposed to temperature variations and even to protect from low pH during gastric absorption (Zhang et al., 2016). Studies regarding the use of diets supplemented with several EOs to minimize the stress caused by handling or air exposure remain scarce. In addition, due to the species-specific effects of EOs, it would be interesting to check the efficacy of these EOs on more fresh- and seawater species.

The use of nanotechnology can potentialize the EO effects. Lecithin-based nanoemulsion of clove oil showed faster anesthesia induction and recovery in goldfish, *Carassius auratus*. Serum glucose was not different, but serum urea was higher in goldfish anesthetized with the nanoemulsion than with free clove oil (Gholipourkanani et al., 2015). The nanoencapsulated EO of *M. alternifolia* exert potent bactericidal action, presenting 100% of curative efficacy in silver catfish infected with *Pseudomonas aeruginosa*, while free EO only showed 70% of curative efficacy (Souza et al., 2017c). Consequently, nanotechnology shows promising results that deserve further analysis.

Medicinal plants have broad antimicrobial properties against important fish pathogens. Further studies on chronic and acute toxicity and on the deleterious effects of herbal medicines on treated organisms, non-target organisms, and the environment are encouraged. Most studies on effectiveness have been based on *in vitro* testing or even conducted under laboratory conditions. Therefore, further practical and economic studies are needed to enable replacement of the current treatments. Joint work between the supply chain, industry, and researchers are paramount to studying medicinal plants that may present antimicrobial effects against important fish pathogens.

## Author Contributions

CS wrote the manuscript and drew the figures. BH wrote from the section “Essential Oils.” BB, JM-S, JM, and MB carried out the corrections on the manuscript.

## Conflict of Interest Statement

The authors declare that the research was conducted in the absence of any commercial or financial relationships that could be construed as a potential conflict of interest.
